# Ionizing radiation abrogates the pro-tumorigenic capacity of cancer-associated fibroblasts co-implanted in xenografts

**DOI:** 10.1038/srep46714

**Published:** 2017-04-25

**Authors:** Maria Tunset Grinde, Jørg Vik, Ketil André Camilio, Inigo Martinez-Zubiaurre, Turid Hellevik

**Affiliations:** 1 Department of Radiology, University Hospital of Northern-Norway, Tromsø, Norway; 2 Molecular & Clinical Inflammation Research Group, Department of Clinical Medicine, University of Tromsø, Norway; 3 Lytix Biopharma, Gaustad Alleen, NO-0349, Oslo, Norway; 4 Department of Radiation Oncology, University Hospital of Northern-Norway, Tromsø, Norway

## Abstract

Cancer-associated fibroblasts (CAFs) are abundantly present in solid tumors and affect tumorigenesis and therapeutic responses. In the context of clinical radiotherapy, the impact of irradiated CAFs to treatment outcomes is largely unexplored. Aiming at improving radiotherapy efficacy, we have here explored the effect of radiation on the inherent pro-tumorigenic capacity of CAFs in animals. Ionizing radiation was delivered to cultured CAFs as single-high or fractionated doses. Tumor development was compared in mice receiving A549 lung tumor cells admixed with irradiated or control CAFs. Biological mechanisms behind tumor growth regulation were investigated by quantitative histology and immunohistochemistry. Viability assessments confirmed that irradiated CAFs are fully functional prior to implantation. However, the enhanced tumorigenic effect observed in tumors co-implanted with control CAFs was abrogated in tumors established with irradiated CAFs. Experiments to ascertain fate of implanted fibroblasts showed that exogenously administered CAFs reside at the implantation site for few days, suggesting that tumor growth regulation from admixed CAFs take place during initial tumor formation. Our work demonstrate that irradiated CAFs lose their pro-tumorigenic potential *in vivo*, affecting angiogenesis and tumor engraftment. This finding propose a previously unknown advantageous effect induced by radiotherapy, adding to the direct cytotoxic effects on transformed epithelial cells.

Solid tumors are complex tissues, consisting of aberrantly growing malignant cells and a cocktail of non-transformed cells that altogether play fundamental roles in cancer sustainability and therapeutic responses^1^,^2^,^3^. Intriguingly, the tight dependency of cancer cells to the stroma in which they grow reveals appealing diagnostic and therapeutic opportunities^4^,^5^,^6^. Carcinoma-associated fibroblasts (CAFs) are a heterogeneous and multifunctional population of mesenchymal cells that function as key drivers of tumorigenesis^7^,^8^,^9^,^10^,^11^. The presence of CAFs in large numbers is generally associated with high-grade malignancy and poor prognosis^12^, but a protective role for CAFs is also suggested^13^. The direct or indirect involvement of tumor-infiltrating CAFs in therapy resistance has long been acknowledged, and recent reports have indicated that secreted factors produced by cells of the tumor microenvironment are interfering negatively with the response of cancer cells to chemotherapy and targeted drugs^14^,^15^. However, in the context of radiotherapy (RT), despite its extended use and impact in cancer management^16^, relatively little knowledge exists regarding the role played by non-malignant stromal cells in patient outcome post-RT^17^. Pre-clinical and clinical studies have suggested that tumor responses to ionizing radiation (IR) may be influenced by the microenvironment^18^,^19^,^20^, especially considering that in clinical settings, all tumor-associated host cells within the *planning target volume* will receive the same prescribed radiation dose as adjacent malignant cells. Hence, to improve cure rates from treatment strategies involving radiotherapy, and to avoid future relapses post-RT, it is vital to uncover the global cellular and molecular responses triggered by RT in all the tumor elements, including contributions effectuated by non-malignant cells^20^,^21^,^22^. Given the fundamental role played by CAFs on general tumor growth regulations^11^,^23^, we hypothesized that CAFs may change their phenotype and pro-malignant nature upon irradiation, and that this circumstance could influence the ultimate fate of tumors post-radiotherapy.

In the past, we have studied the effects of ionizing radiation on primary CAF cultures directly isolated from lung tumor specimens. Despite displaying a prominent radio-resistant phenotype, exposure of primary human lung CAFs to single high-dose RT induces permanent DNA-damage responses and irreversible senescence, promotes enhancement of integrin-mediated focal contacts^24^, and alter the release of certain cytokines and growth factors^25^. In the latter study, effects of CAF-conditioned media on tumor cell growth and migration was also explored, but no significant differences were observed. In a more recent study we have also shown that radiation exposure do not alter the immunosuppressive abilities of CAFs^26^. In this work, we aimed at expanding our knowledge by studying the impact of admixed irradiated CAFs on the fate of tumors grown in xenografts. Results from this study suggest that CAFs lose their pro-tumorigenic capacity after radiation exposure. Such findings uncover a previously unknown beneficial effect elicited by radiotherapy besides the direct killing of malignant cells.

## Results

### CAFs survive single-high dose and fractionated radiation doses

**Figure 1:**
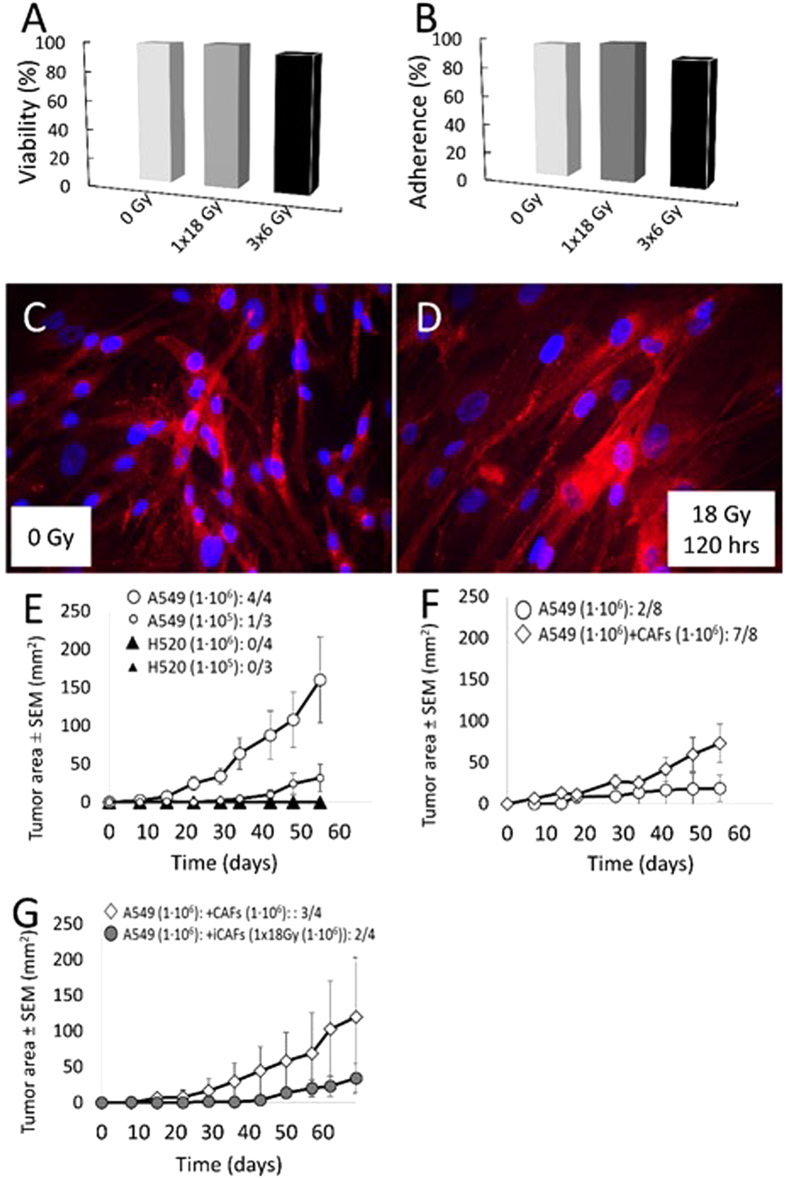
Viability of irradiated CAFs & Tumor growth characteristics. Viability (**A**) and plating efficiency (**B**) of cultured irradiated CAFs, prior to implantation. In (**A**), viability was measured 24 hours after last radiation dose. In (**B**), plating efficiency of CAFs also 24 hours post-IR. Columns in A and B show values from one CAF donor and are representative outcomes from three different donors. In (**C**,**D**) fluorescent micrographs of cultured human lung CAFs stained with anti-human PDGFRα antibody (red) and nuclear DNA (DAPI, blue). Panel (**D**) shows PDGFRα-staining of CAFs that were irradiated (1 × 18 Gy) and fixed 5 days post-IR. Panels (**E**–**G**) demonstrate tumor growth curves for xenografts established s.c. in athymic nude mice in pilot experiments. In (**E**), tumor take and growth from two different human lung tumor cell lines, A549 and H520, was compared. In (**F**), tumor growth characteristics of bilateral tumors established with tumor cells vs. tumor cells/CAFs. Left flank was inoculated with only A549 cells (1 × 10^6^), whereas right flank received a mix of A549 cells (1 × 10^6^) and CAFs (1 × 10^6^). In (**G**), comparative tumor growth kinetics in animals inoculated with admixed control CAFs or irradiated (1 × 18 Gy) CAFs. CAFs were irradiated 24 hours prior to implantation. Tumor area: *A* = *π·l·b*.

Initial experiments were conducted to ascertain if the radiation schemes used for the *in vivo* experiments were critically affecting viability or performance of CAFs. Experimental settings were identical to the ones used later on for the *in vivo* trials prior to implantation. These preliminary tests confirmed that viability of CAFs (from 3 different donors) was comparable to non-irradiated controls, when measured 24 hours post-irradiation by Trypan blue exclusion assay (Fig. 1A). Similarly, the large majority of living cells from both irradiated and non-irradiated cultures were able to adhere to plastic after cell detachment, demonstrating good performance of the irradiated cells in terms of cell adhesion and spreading prior to implantation (Fig. 1B). Phenotypically, both control and irradiated CAF cultures showed abundant and homogenous expression of the fibroblast marker platelet-derived growth factor-receptor-α (PDGFRα) (Fig. 1C,D). The human specific antibody against PDGFRα was used in subsequent animal experiments to trace implanted CAFs.

### Radiation abrogates the enhanced tumor-growth effects exerted by admixed CAFs

**Figure 2:**
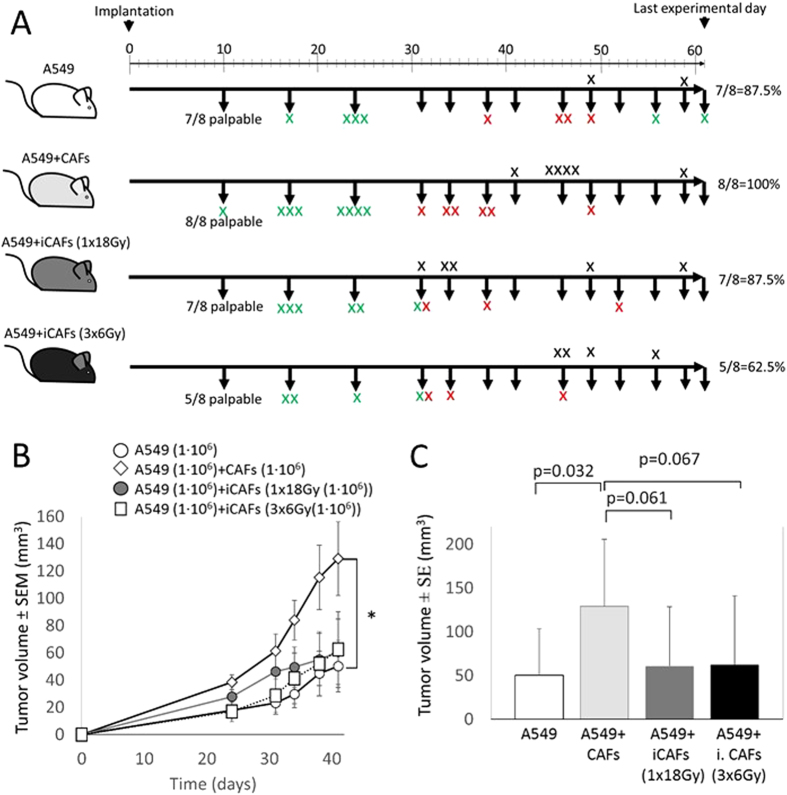
Tumor take & Tumor growth kinetics in xenografts. Monolateral xenograft tumors were established s.c. in athymic Balb/c nu-nu mice. (**A**) Timeline reflecting tumor take and growth kinetics between experimental groups. Green 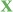 indicates time-points when tumors reached 4 mm; Red 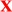 reflects time-points when tumors reached 7 mm; Black **X**: reflects tumors retrieved due to ulceration or with diameters ≥10 mm. (**B**) Tumor growth kinetics: (○) A549 lung tumor cells (n = 7), (◊) A549 tumor cells with control CAFs (n = 8), (●) A549 tumor cells with irradiated CAFs (iCAFs) (1 × 18 Gy) (n = 7), (▪) A549 tumor cells with fractionated-irradiated CAFs (3 × 6 Gy) (n = 5). (**C**) Tumor volumes at 41 days post-implantation. Group 1 (n = 7); group 2 (n = 8); group 3 (n = 7); group 4 (n = 5). Differences in tumor volumes between group 1 and 2 were statistically significant (*p < 0.05), and borderline significant between group 2 and groups 3 or 4 (p = 0.061 and 0.067, respectively).

A fundamental part of this study was to determine if CAFs - upon exposure to ionizing radiation - maintain their well described tumor promoting effects when co-implanted with tumor cells. A series of pilot experiments in animals were therefore conducted to decide on the most optimal parameters for experimentation, such as most suitable tumor cell line, most suitable number of inoculated cells, rate of tumor growth with and without CAFs and best implantation strategy (monolateral vs. bilateral) (Fig. 1E–G). Chosen parameters for the main experiment are described in the methods section. Under such conditions, A549 lung cancer cells (1 × 10^6^) injected either alone or admixed with non-irradiated or irradiated CAFs (1 × 10^6^) were able to establish subcutaneous tumors in the flank of athymic mice. In the first large experiment (Fig. 2), four experimental groups were included (8 mice/group), comprising **1)** A549 lung tumor cells alone, **2)** A549 admixed with non-irradiated CAFs, **3)** A549 admixed with single-high dose irradiated CAFs (1 × 18 Gy) and **4)** A549 admixed with fractionated-irradiated CAFs (3 × 6 Gy). Five to ten days after implantation, palpable tumors were observed in the majority of the animals (Fig. 2A). Tumor growth curves representing two months of incubation are shown in Fig. 2B. Tumors were first observed in mice that received A549 tumor cells admixed with non-irradiated CAFs (group 2), indicating both improved tumor take (to 100%) and accelerated tumor growth in this group compared to the other groups (Fig. 2A,B). Additionally, A549 tumor cells co-implanted with control CAFs established tumors earlier, more frequently and with enhanced tumor growth kinetics (2–3 fold enhancement, p < 0.05) compared to tumors established exclusively with A549 tumor cells. Interestingly, tumor growth was slower in animals implanted with A549 and irradiated CAFs (groups 3 and 4), compared to tumor growth in animals receiving a mix of A549 cells and non-irradiated CAFs (group 2) (p < 0.05) (Fig. 2B). Another radiation-induced effect observed in the group with co-implantation of *fractionated*-irradiated CAFs (A549 + f-iCAFs; 3 × 6 Gy) was reduced tumor-take (62.5%) compared to the control group (only A549)(87.5%) (Fig. 2A). Nevertheless, growth kinetics for these two groups were comparable (Fig. 2B).

Tumors were collected at endpoint (day 61), or when tumor diameters reached 10 mm. Some animals were sacrificed before the experimental endpoint due to necrosis/ulceration of tumor lesions. Hence, tumor volumes measured at day 41 post-implantation were largest in mice receiving A549 together with non-irradiated CAFs, whereas tumor-sizes in groups 3 and 4, containing irradiated CAFs, were comparable to tumors established with only A549 (Fig. 2C). Of note, metastatic lesions in lung or bone were not observed in any mice.

### Mechanisms behind iCAF-mediated tumor growth regulation revealed by histopathology

**Figure 3:**
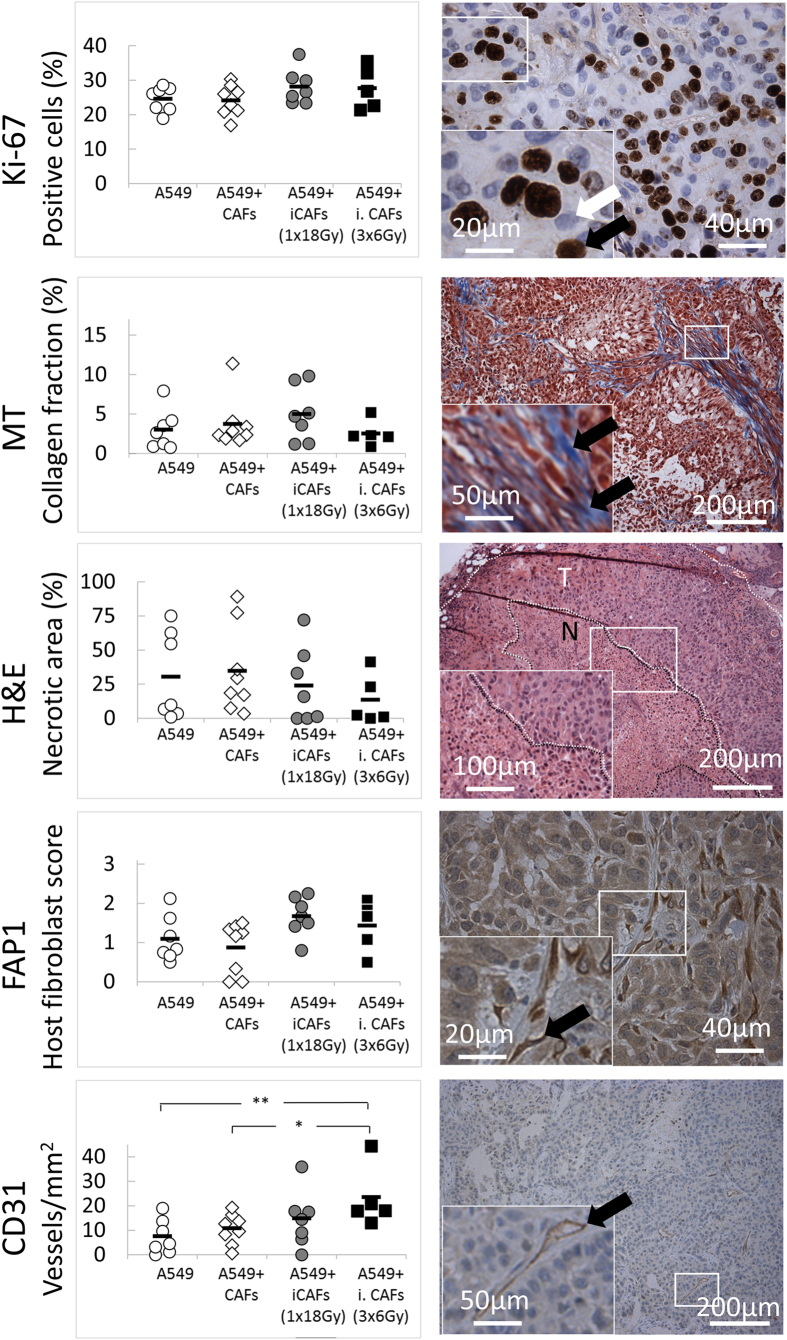
Quantitative assessments of tumor biological markers by histopathology. Tissue sections from tumor xenografts harvested 41–61 days post-implantation were stained for Ki-67 (proliferation), Masson’s trichrome/MT (collagen), H&E (necrosis), FAP-1 (human/murine CAFs), and CD31 (endothelial cells). Left panels; software-based quantitative evaluation of staining. Each dot represent the average value of multiple images taken from same tumor (one dot/animal). Right panels show representative images of the different markers taken from randomly selected tumors.

Aiming at defining potential mechanisms behind the CAF-mediated tumor growth regulations, we performed quantitative histology and immunohistochemistry analyses of major tumor biological markers, including proliferation rate, extent of necrosis, angiogenesis, matrix deposition, and tumor infiltration by inflammatory cells and fibroblasts. Software-based quantitative determinations of Ki-67 (proliferation), hematoxyline and eosin (H&E) (necrosis), and Masson’s trichrome (MT) (connective tissue/collagen) revealed no significant differences between experimental groups (Fig. 3). Ki-67 staining demonstrated that (20–35)% of tumor cells were in a proliferating phase, whereas Masson’s trichrome staining of collagen revealed that connective tissue in tumors represented (0–10)% of total tumor area. H&E-staining visualized large variability in extent of tumor necrosis, ranging from 0% to near 100% of total tumor area. Of note, large variability in necrosis and collagen deposition in tumors within the same experimental groups was observed.

Intriguingly, quantification of microvessel-density by the endothelial cell marker CD31 revealed that tumors grown in the presence of irradiated CAFs had enhanced micro-vessel formation, relative to tumors grown without CAFs (p = 0.009) or with non-irradiated CAFs (p = 0.029) (Fig. 3, bottom panels). Additionally, tumors established with fractionated-irradiated CAFs (3 × 6 Gy) demonstrated higher microvessel density than CAFs exposed to a single-high dose of radiation, albeit the differences were not statistically significant between these groups. Staining of tumors with the murine fibroblast marker^27^ fibroblast activation protein-1 (FAP-1) revealed substantial infiltration of host fibroblasts in most tumors. Although significant differences were not observed between experimental groups, we identified a trend towards increased numbers of FAP-1^+^ cells (i.e. host fibroblasts) in tumors established with irradiated CAFs (1 × 18 Gy) relative to tumors established with only cancer cells.

**Figure 4:**
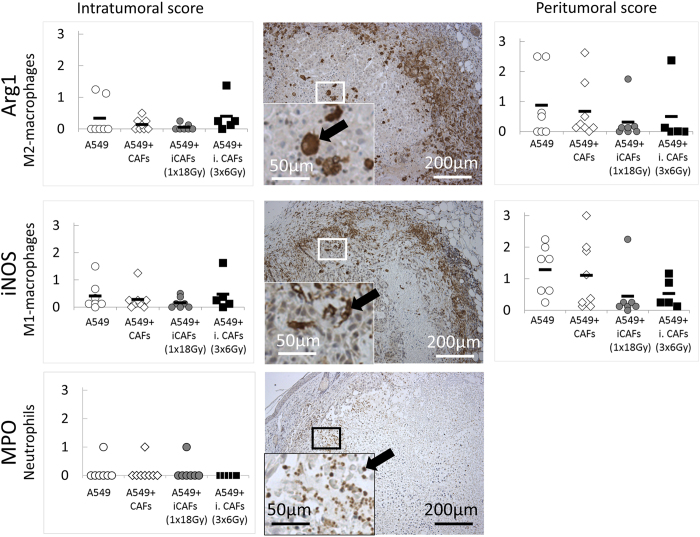
Assessments of inflammatory cell infiltration in tumors harvested at late stages. Tissue sections from tumor xenografts harvested at end-point of study, i.e. 41–61 days post-implantation of cells, were immune-labelled for iNOS (M1-macrophages), Arg1 (M2-macrophages) or MPO (neutrophils). Intratumoral and peritumoral scores in left and right panels, respectively. Middle panel; representative images from each staining from randomly selected tumors.

The extent of inflammatory reactions and immune cell infiltration was studied by the use of specific markers that identify inflammatory/immune cells, including inducible nitric oxide synthase (iNOS) to detect type-I (M1) macrophages, arginase-1 (Arg1) to detect type-II (M2) macrophages and myeloperoxidase (MPO) to detect neutrophils. To increase the accuracy in evaluating these markers, we divided the scorings into peritumoral and intratumoral marker expression (Fig. 4). Tumors from all experimental groups were to some extent infiltrated by murine immune cells, including macrophages and neutrophils, as shown in Fig. 4. However, most of the late-stage tumors revealed low levels of intratumoral M2-macrophages (Arg1^+^; (0–5)%) and M1-macrophages (iNOS^+^; (0–1)%). Peritumoral presence of macrophages (M1 and M2) had a wide range of values, reaching >50% in some tumors, but most tumors had a lower score (<5%). For neutrophils, all, except three tumors, were completely negative for MPO (infiltration <5% of tumor area). Once again, substantial variations in scoring rates for immune markers were observed within each experimental group. Finally, the presence of implanted CAFs in tumors was checked by human-specific anti-PDGFRα antibody. Importantly, we could not identify implanted CAFs (or CAF remnants) in tumors in any of the experimental groups.

### Fate of implanted fibroblasts in xenografts

**Figure 5:**
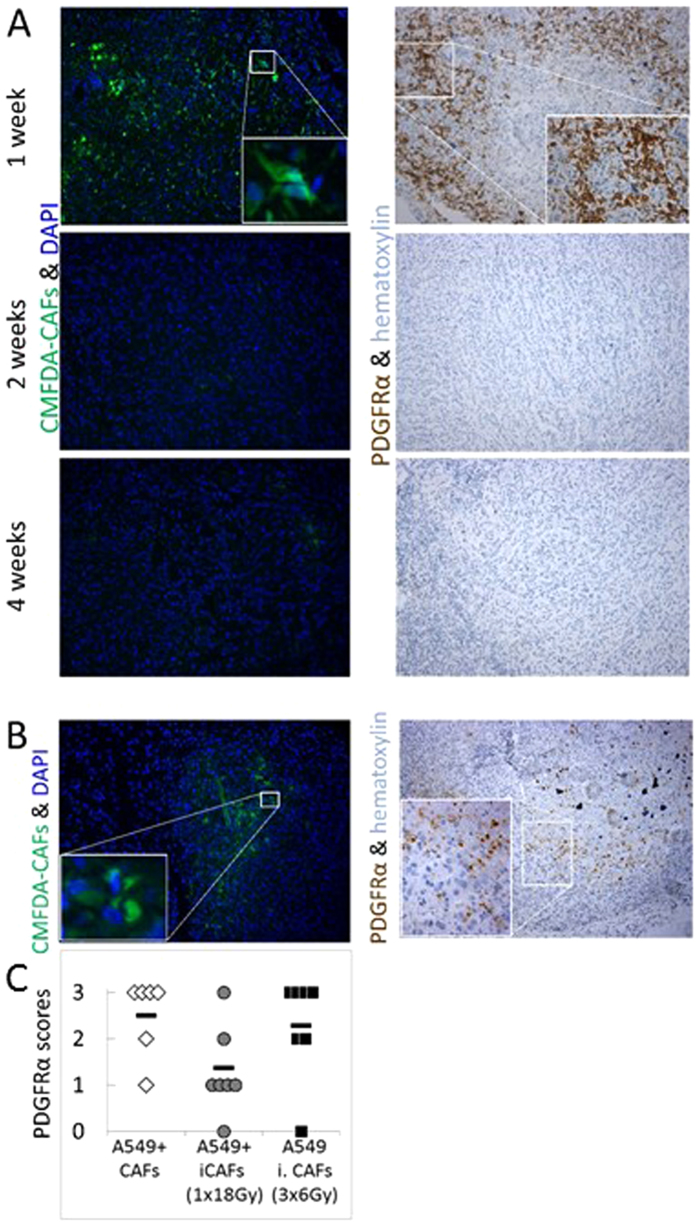
Fate of human CAFs co-transplanted in A549 xenografts. (**A**) Time-course experiment showing presence/absence of implanted human CAFs in tumors harvested at 1, 2 and 4 weeks post-implantation. (**B**) Detection of irradiated CAFs (1 × 18 Gy) in tumors 1 week post-implantation; Exogenously administered CAFs are visualized by green CMFDA fluorescence (left panels) and by immune-staining against human-specific anti-PDGFRα antibody (right panels). (**C**) Blinded quantitative scorings of PDGFRα-positive fibroblasts in early tumor tissues with admixed CAFs.

The latter observation prompted us to explore the fate of transplanted CAFs in our model, and to elucidate if non-irradiated CAFs, in contrast to irradiated CAFs, undergo cell expansion *in vivo*. We conceived a double approach to answer this question, based on **a)** permanent intracellular fluorescence labelling of cultured CAFs by the fluorochrome 5-chloromethylfluorescein diacetat (CMFDA); and **b)** detection of human CAFs in tumor tissue by a human-specific anti-PDGFRα antibody (Fig. 5A). Tumors were collected at various time-points post-implantation, including 1 week (earliest macroscopic manifestation of tumors), 2 weeks and 4 weeks (tumor diameters ~5 mm). Results from this experiment confirmed that in our tumor model, *transplanted* human CAFs were present at early stages after implantation (first week), but were not traceable in specimens collected 2 and 4 weeks after implantation. In some tumors, labelled cells had a spindle-shaped or fibroblast-like morphology (within healthy tumor areas) while in other tumors the labelled cells displayed a more round morphology (normally associated to tumors with extended necrosis). Likewise, the presence of *irradiated* CAFs was also checked in tumors collected at early time-points post-implantation (Fig. 5B). We confirmed that irradiated CAFs were indeed present at the implantation site during the first week after inoculation of cells. The final fate of transplanted CAFs is uncertain, but based on the frequently observed apoptotic-like round morphology of labelled cells, and the presence of abundant subcellular remnants, we speculate that most implanted CAFs might gradually die *in situ* some few days after implantation. To validate the successful transplantation of irradiated CAFs, we quantified human CAFs based on PDGFRα expression in one-week-old tumors (Fig. 5C). Results from these measurements show that CAF transplantation was successful in all experimental groups. The relative lower numbers of human CAFs in the group of tumors established with CAFs exposed to a single-high (1 × 18 Gy) dose of radiation could be explained by radiation-induced senescence^24^ and the consequent loss of cell division in comparison to the two other groups. Of note, the control group and the fractionated-irradiated (3 × 6 Gy)-group demonstrated comparable numbers of transplanted CAFs.

### Tumor biological features examined by histopathology at early stages of tumor growth

**Figure 6:**
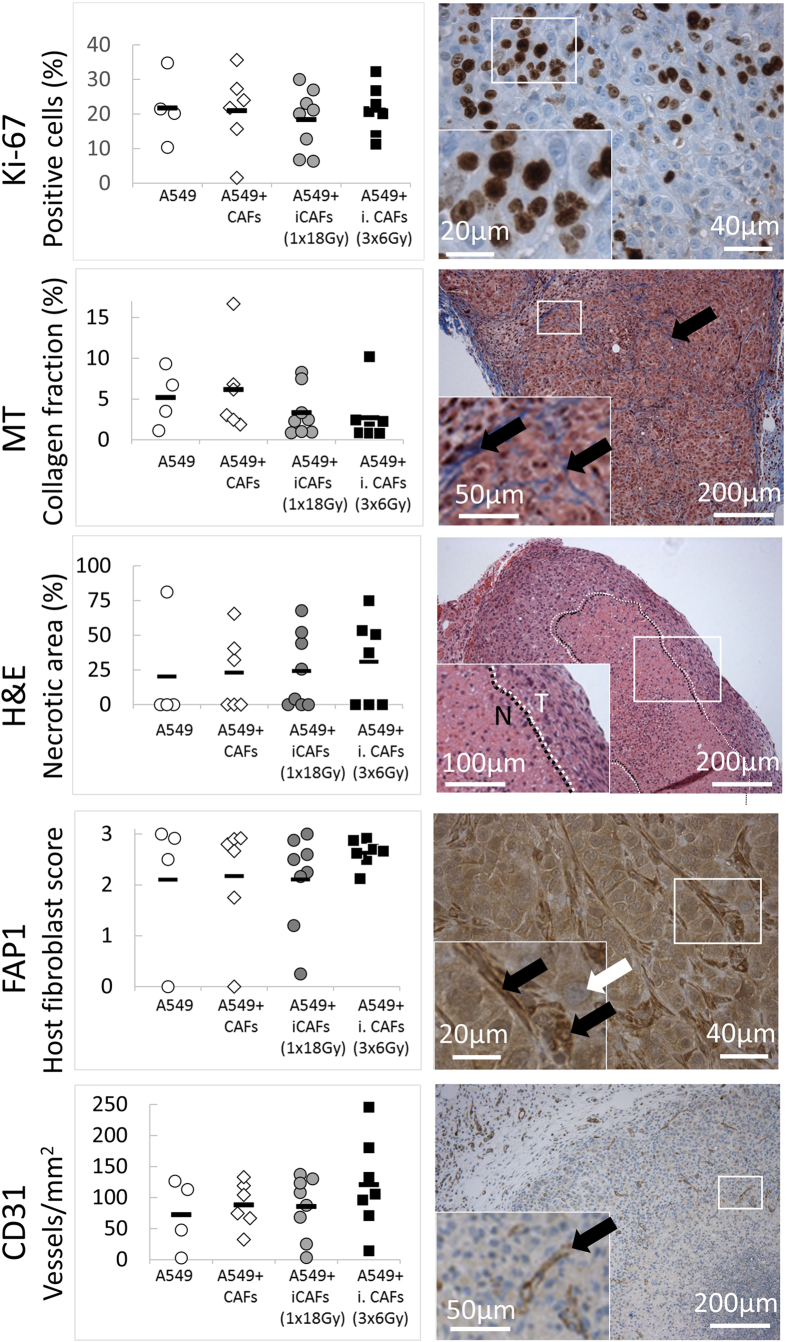
Tumor biological markers in early-stage xenografts. Tissue sections from tumor xenografts harvested 7–10 days post-implantation were stained for Ki-67 (proliferation), Massons trichrome/MT (collagen deposition), H&E (necrosis), FAP-1 (human/murine CAFs), and CD31 (endothelial cells). Left panels show quantitative determinations of markers, one dot per animal. Right panels show representative images of the different markers taken from randomly selected donors.

**Figure 7:**
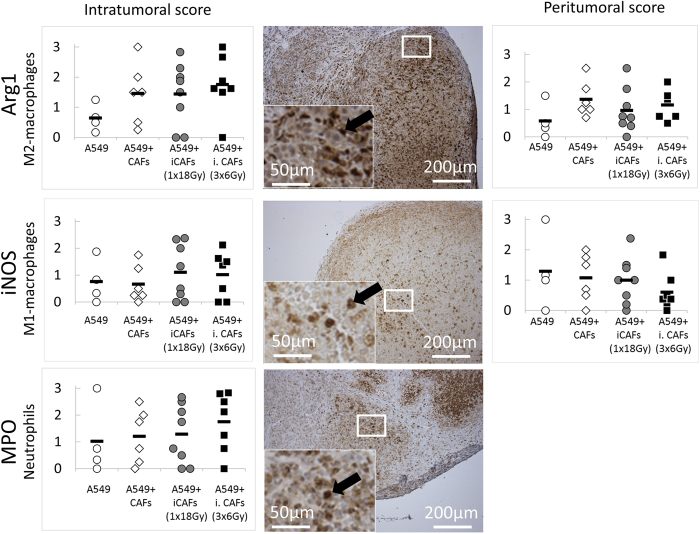
Immune-specific markers in early tumors. Histology of tumor xenografts harvested at early stages of tumor growth (7–10 days post-implantation). Tumor tissue sections were labelled with antibodies against iNOS (M1-macrophages), Arg-1 (M2-macrophages) and MPO (neutrophils). Intratumoral and peritumoral scores shown in left and right panels, respectively. Middle panel shows representative images of the different markers taken from randomly selected donors.

Since admixed CAFs (both irradiated and non-irradiated) were only present at early stages of tumor formation, we decided to check again for relevant tumor biological features in tumors collected immediately after becoming macroscopically detectable (7–10 days post-implantation). Thus, a similar main experiment as the one carried out initially was performed, with the only exception that tumors were collected shortly after implantation. Twenty-five small tumors (of 32 inoculated animals) could be collected when tumors became palpable. The same list of tissue markers used in the previous experiment were examined in early-stage tumors. In Fig. 6 (tumor biological markers) and Fig. 7 (immune/inflammatory cells), dot plots with means for each group are presented. In general, the new analyses in early-formed tumors revealed comparable results as in late-stage tumors, showing analogous differences between groups for basically all the tested markers. Unfortunately, the variance of values within groups kept being quite high also in early-collected tumors. Regarding microvessel density, a similar trend as in late-stage tumors was found; marked by increased values in tumors from the fourth group (A549 + iCAFs; 3 × 6 Gy) (Fig. 6). The results were however not significant (p = 0.075). Of note, IHC staining demonstrated that xenografts at early time-points were extensively infiltrated by host immune cells, including macrophages and neutrophils, as shown in Fig. 7. However, intratumoral and peritumoral infiltration was highly variable within groups for all tested immune markers. Importantly, by comparing histopathological scores between early- and late-stage tumors, some clear differences were observed. For instance, the number of vessels per area (detected by CD31-stains) was about (5–10)-fold elevated in early stage versus late stage tumors (Figs 3 and 6, bottom panels). Moreover, macrophages and neutrophils were more abundantly present in early stage compared to late stage tumors.

## Discussion

In the context of clinical radiotherapy, both cancerous cells and the rest of tumor-associated host cells receive the same prescribed radiation dose. Under injurious circumstances such as those instigated by radiotherapy, fibroblasts are probably one of the most resistant cell types and could therefore participate towards tumor relapse. Hence, the aim of this study was to evaluate the potential tumor growth regulatory effects exerted by irradiated CAFs *in vivo*. The experimental strategy comprised subcutaneous implantation of tumor cells admixed with irradiated or non-irradiated CAFs in athymic mice. Here we demonstrate that, at least in this xenograft model, CAFs lose their natural pro-tumorigenic properties upon exposure to ionizing radiation. This outcome represents a gainful effect exerted by radiotherapy that has not been described hitherto.

To unveil the contribution of CAFs in tumor development and therapeutic outcomes, researchers have relied on preclinical tumor models that include co-implantation of different cell types. In our model, admixed CAFs clearly enhance engraftment and tumor growth compared to engraftment with tumor cells only. Several studies have demonstrated that co-injection of Matrigel^28^ or normal fibroblasts along with tumor cells^29^,^30^,^31^,^32^,^33^ is able to bypass the tedious process of stromal cell recruitment and activation^34^, resulting in improved tumor take and accelerated tumor formation^32^. However, in some animal models of breast^35^,^36^ and prostate cancers^37^, it has been demonstrated that admixed CAFs constitute a rate-limiting factor for tumor progression^23^, exerting even more powerful tumor enhancing effects than normal fibroblasts or Matrigel^32^. The latter fact highlights the importance of the phenotypic differences between quiescent/normal and activated fibroblasts^10^,^31^,^35^,^38^.

Among the possible mechanisms behind CAF-mediated tumor growth regulation *in vivo*, it has been frequently observed an enhancement of angiogenesis during early phases of tumor formation via secretion of miscellaneous CAF-derived factors, including SDF-1/CXCL12^35^, CXCL14^39^, FGF-2^40^,^41^, PDGF^42^, IGF2^43^, TGFβ^44^,^45^ or CTGF^46^. Alternative suggested mechanisms are increased deposition of basement membrane components in tumors^30^, expression of fibroblast activation protein (FAP-1)^47^, metalloproteinase 2 (MMP-2)^48^ or cofilin-1 and IL-6^49^. In addition, CAFs are able to directly modulate the recruitment, polarization and function of innate immune cells via secretion of cytokines and chemokines such as CCL2, CXCL1 or CXCL14^50^. In this study, we have analyzed angiogenesis, inflammation and other major tumor biological features by histology and immunohistochemistry. In tumors retrieved at late stages of growth we have not observed significant differences in the extent of angiogenesis between tumors formed by only tumor cells or admixed with control CAFs. Other essential parameters such as desmoplastic reactions, tumor cell proliferation or inflammatory cell infiltration also remained comparable between experimental groups. Nevertheless, it is important to consider that the intragroup variability found for most markers in our model was considerably high and could have influenced the statistical significance of the results.

The lack of significantly relevant CAF-mediated effects on tumor biological parameters at late stages of growth prompted us to explore the fate of CAFs following implantation. Indeed, certain controversy exists on to which extent implanted human CAFs persist at the injection site during tumor development. Some authors claim that CAFs have left the injection site already 13 days after being co-implanted^41^, whereas others have reported that CAFs are found in tumors even 30 days post-injection^35^. Nonetheless, the majority of laboratories that have studied this particular aspect coincide on the view that regardless of tumor model or implantation site used, exogenous fibroblasts only reside at the injection site during early stages of tumor formation^41^,^48^,^51^,^52^,^53^. Our model faithfully reproduce the same scenario observed by many others, where human CAFs become untraceable already 2 weeks post-implantation, and tumors become instead infiltrated by host fibroblasts. This observation allows thinking that implanted human CAFs exert their effects at very early stages of tumor formation, apparently affecting aspects related to tumor cell engraftment, early tumor cell survival, early angiogenesis or early inflammatory reactions. Because of the last mentioned observation, we repeated the experiments, but collected tumors early, i.e. when neoplasms were barely visible (7–10 days post-implantation). In line with what was expected, levels of neo-angiogenesis and rate of inflammatory cell infiltration was higher in early than late collected tumors, coinciding with onset of local inflammatory reaction after cell transplantation. However, we did not find statistically significant differences in tumor biological features that could explain increased tumor cell engraftment in animals receiving tumor cells and control (non-irradiated) CAFs.

The most relevant finding in our study is the observation that CAFs lose their pro-tumorigenic effects after exposure to ionizing radiation, given as a single-high dose (1 × 18 Gy) or oligo-fractionated doses (3 × 6 Gy). We will emphasize that the irradiated CAFs used in this study were alive and functional at the time of implantation. In fact, other scholars in the field have previously demonstrated that the fibroblast is a highly radioresistant cell type, and that fibroblasts established in culture dishes may survive single doses above 30 Gy^54^,^55^,^56^. The effect of ionizing radiation on fibroblasts, both on normal and cancer-associated fibroblasts, has been studied to some extent *in vitro*. Of interest, simultaneous irradiation of normal fibroblasts and murine (adenocarcinoma) lung tumor cells in co-culture reportedly was abrogating the pro-migratory phenotype of these carcinoma cells^57^. Others have performed transcriptome studies, comparing irradiated and non-irradiated fibroblasts, and have thus revealed profound changes in biological functions and processes involved in DNA repair, activation of stress responses, cell cycle arrest, senescence-associated genes, autophagy regulatory elements, ROS production and immune responses^54^,^58^,^59^. Our group has previously shown that irradiated lung-CAFs become prematurely senescent (80% rate by 1 × 18 Gy, 50% rate by 6 × 3 Gy), slow down their proliferative rate, and display reduced migratory function^24^. Most importantly, the pattern of CAF-secreted proteins comprising numerous inflammatory mediators, extracellular matrix proteins, proteases, growth factors, chemokines and cytokines become significantly altered after irradiation^24^,^25^. Studies on CAF responses to IR have unveiled intriguing changes in the secretion of paracrine signal molecules, including enhancement of bFGF and reduction of CTGF and SDF-1^25^. In this study, we do not see clear changes in the patterns of ECM deposition, host fibroblast recruitment or tumor cell proliferation. However, we do observe enhanced neo-angiogenesis in tumors formed with admixed *fractionated*-irradiated CAFs, both at early (1.7-fold) and late (3.3-fold) tumor formation. This observation is in agreement with previous analyses of the iCAF secretome, demonstrating increased release of bFGF and decreased release of the anti-angiogenic factors thrombospondin-1 and -2 after radiation exposure^25^. Interestingly, the presence of implanted CAFs, and in particular *fractionated*-irradiated CAFs, triggered enhanced infiltration of macrophages and neutrophils in early tumors. Microvessel density – both at early and late tumor formation - displayed a similar trend, with maximal vessel density in tumors initiated with fractionated-irradiated CAFs. Nevertheless, the enhanced angiogenesis in the irradiated-CAF groups may be interpret as a controversial finding, since the tumor volume of xenografts initiated with admixed irradiated CAFs is actually reduced. We speculate that in our mouse model, the process of vessel formation and development of functional vasculature in tumors is inefficient and therefore, early enhancement of endothelial cell infiltration does not support eventually enhanced tumor growth. In fact, vessel density parameters were considerably lower in tumors collected at late compared to early stages of growth.

The available literature on tumor growth regulations by irradiated fibroblasts *in vivo* is scarce. In this context, it is utterly important to consider that most available data comes from models that comprise normal tissue fibroblasts or immortalized fibroblast cell lines instead of primary cultures of reactive^10^,^60^ tumor-associated fibroblasts^51^,^56^,^61^. Some of the referred studies do not focus on radiation effects *per se*, but rather aim at exploring cellular senescence, using radiation as an experimental tool for senescence induction^56^,^62^. With this background in mind, other laboratories have demonstrated that at radiation doses exciding 1 × 10 Gy, the phenotype of fibroblasts become altered, with induction of irreversible DNA-damage responses and development of stress-induced senescence^24^,^62^. In the cancer setting, senescent fibroblasts play deleterious roles by contributing to chronic inflammatory reactions, promoting angiogenesis and nourishing growth and invasion of transformed epithelial cells^56^,^62^. In our study, we observe opposite effects. The irradiated lung CAFs we have used here turn senescent several days after IR exposure^24^, however, they lose their tumor-supporting characteristics, with a demonstrated prolonged latency, reduced tumor-take and retarded tumor growth. Our tissue examinations were insufficient to explain the mechanisms behind the observed effects. Of note, in tumors collected at early stages, we observed a reduced number of implanted CAFs in the experimental group receiving admixed CAFs pre-treated with a single-high radiation dose (1 × 18 Gy) (Fig. 5). We cannot exclude the option that an accelerated cellular decay of CAFs receiving such high radiation dose could be the reason behind the reduction in tumor promotion. In such case, CAF-derived effects would not be attributable to radiation-induced phenotypic changes, but plainly to increased cell death. On the contrary, we observe comparable numbers of transplanted CAFs when comparing the non-irradiated group and the (3 × 6 Gy)-irradiated group, the latter group clearly showing a significant reduction in tumor promotion. In this case, the loss of tumor enhancing effects is likely attributable to radiation-induced phenotypic changes in CAFs. Importantly, other authors have also questioned the tumor-enhancing nature of irradiated or senescent fibroblasts^33^,^57^,^61^,^63^.

In conclusion, in this study we unveil a new advantageous effect elicited by radiotherapy, which adds to the direct killing of transformed epithelial cells, in the form of silencing the natural tumor-promoting actions of CAFs. Analyses of crucial tumor biological features by histopathology, combined with earlier knowledge about radiation-mediated effects on CAFs were not sufficient to address the mechanisms behind the observed tumor growth regulation. Further studies using perhaps other tumor cell lines and more sophisticated animal models - such as orthotopic or patient-derived xenografts or genetically engineered mouse models - will be needed to make measurable improvements in our understanding on the contribution of CAFs to radiotherapy outcomes.

## Materials and Methods

### Lung cancer cell lines

Human lung cancer cell lines A549 (lung adenocarcinoma) and NCl-H520 (squamous cell carcinoma) were purchased from LGC Standards AB (Borås, Sweden). Cells were cultivated in a humidified incubator at 37 °C, containing 5% CO_2_ and 20% O_2_. Incubation medium consisted of RPMI-1640 supplemented with 10% heat deactivated fetal bovine serum (FBS), L-glutamine and penicillin (1%) and streptomycin (1%).

### Human material, CAF isolation and cultures

Human lung CAFs were isolated from freshly resected non-small cell lung carcinoma (NSCLC) tumor tissue from patients undergoing surgery at the University Hospital of Northern Norway, Tromsø. Lung tumor specimens from four different donors were included in the study. The Regional Committee for Medical and Health Research Ethics (REK-Nord) approved the use of human material for this study (Project-ID: 2009/895-4), and informed written consent was obtained from all patients. All methods involving human material were performed in accordance with relevant guidelines and regulations. Upon surgical resection of tumors, fibroblasts were isolated by the outgrowth method, as previously described^24^. Mesenchymal-like cells were selected by differential cell detachment, and further expanded in serum-containing incubation medium. CAFs were characterized for the presence of lineage specific markers at the third passage, using flow cytometry and immunocytochemistry^24^. Isolated CAFs were used for experimentation after third and fourth passage (3–4 weeks-old cultures).

### Irradiation of cells

Adherent CAF cultures prepared in T-75 flasks were irradiated when 70–90% confluent with high-energy photons, using a clinical Varian linear accelerator, as previously described^24^. Ionizing radiation was delivered to cell cultures either as a single-high dose (1 × 18 Gy) or as fractionated exposures (3 × 6 Gy) in 24 h intervals. Standard parameters for dose delivery was depth 30 mm, beam quality 15 MV, dose rate of 6 Gy/min and field sizes of 20 × 20 cm. The (last) dose of radiation was given 24 hours before injection of cells into mice. Thermo-Luminescent Dosimeters (TLDs) were applied to confirm dose-delivery within an acceptable ±4%.

### Viability and plating efficiency assays

CAFs from the same donors as those used in the animal experiments were irradiated in the same way as cells utilized for *in vivo* studies, and 24 hours post-irradiation examined for viability and adherence capacities. For that purpose, twenty-four hours after the last radiation exposure, cells were incubated with Enzyme-Free Cell Dissociation Solution (Merck Millipore), then detached and spun down (400 g, 3 min). Resulting cell pellets were dissolved in 500 μL cold medium and kept on ice for 45 min to mimic the procedure for cell implantation. Cell viability was determined by Trypan blue exclusion assay, and percentage viable cells was estimated by dividing number of cells without dye against total number of cells. For analysis of plating efficiency, the harvested cells were transferred to a T-25 flask and incubated further (24 hours, 37 °C) for cell adherence. After incubation, non-adherent cells was counted, and plating efficiency (%) was calculated in relation to total sum of adherent and non-adherent cells.

### *In vivo* xenograft model

Female nude athymic mice (NU/NU Nude Mouse Crl:Nu-Foxn1nu, age 6-7 weeks), weighing 23.3 ± 2.0 g were purchased from Charles River (Sulzfeld, Germany), and acclimatized in the local animal facility for minimum five days before experimentation. All procedures and experiments involving animals were conducted according to regulations by the Federation of European Laboratory Animal Science Association (FELASA) and was approved by the National Animal Research Authority (permission ID 6373, 6942 and 7873). All cells used for implantation were tested for pathogens by Charles River Laboratories International Inc. (Human Comprehensive test, Charles River). Pilot trials were conducted to ascertain the most suitable lung tumor cell line for xenograft formation (between A549 and H520) and optimal amount of implanted cells to achieve stable tumors. For the main experiment, mice received tumor cells alone (A549, 1 × 10^6^ cells/mouse), or tumor cells admixed with (Sham)-irradiated CAFs (1 × 10^6^ cells/mouse). In the second large experiment, for checking tumor features at early stages, we used a tumor cells/CAFs ratio of 2:1 in order to increase the rate of tumor take in animals from the irradiated groups. Cells utilized for implantation were prepared in culture medium (RPMI + 10% FBS) and injected s.c. into the right flank of animals (50 μL/mouse). Tumors were measured twice per week using a digital caliper, and tumor volumes were calculated (
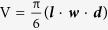
). In the experiment dedicated to tumor growth kinetics analyses, animals were sacrificed when tumors reached diameters of 10–12 mm or, eventually, at the experimental endpoint (day 61). Some animals were euthanized before endpoint of experiment due to animal welfare (necrotic tissue in tumor (N = 7), necrotic tail (N = 1), other reasons (N = 1). In the second experiment, which focused on early tumor formation, tumor tissues were harvested when tumors became macroscopically visible and palpable.

### Histopathology and immunohistochemistry (IHC)

Medium and small-sized tumors were directly immersed into ice-cold 4% paraformaldehyde (PFA) in PBS upon excision, whereas large tumors were perfusion-fixated *in situ* with 4% PFA, excised and stored (24–48) h at 4 °C in 2% PFA/PBS before paraffin-embedding. Tumor tissues were thereafter cut into thin sections (5 μm) with a cryostat, and sections deparaffinized and rehydrated prior to antibody labelling. Necrotic areas were identified in slides stained with hematoxyline and eosin, whereas interstitial collagen depositions were visualized by Masson’s trichrome staining. Cellular proliferation (Ki-67), microvessel density (CD31) and immune cells infiltration were identified by immunostaining. Antibodies utilized for immunohistochemistry analyses are presented in Table 1. Antigen retrieval was mediated by heating sections in citrate buffer (10 min, 95 °C). IHC staining was executed by applying a rabbit specific HRP/DAB detection kit (Abcam, ab64261, UK). For the specific detection of Ki-67, secondary staining was carried out using the UltraView Universal DAB Detection Kit (Roche, 760–500), and performed on a BenchMark XT or BenchMark Ultra platform (Ventana Medical Systems, Arizona, US).

### Image acquisition and quantitative analyses

Image analysis was performed in a blinded manner, by masking the identity of sections prior to image acquisition. Multiple images per tumor specimen (1 to 8, depending on tumor size) were acquired with a Leica CTR6000 microscope (Leica Microsystems, Germany) equipped with a Leica DFC320 digital color camera. Images were collected in a predetermined systematic pattern, with a random starting point. Tissue sections stained for FAP1 and Ki-67 were imaged at 400X, whereas sections stained with H&E and Masson´s trichrome as well as CD31, iNOS, Arg-1 and MPO antibody-labelled sections were imaged at 100X. Quantitative scores per mouse represent average values based on all images collected from a single tumor. The plugin Immunoratio for ImageJ was used for computer-assisted analysis of Ki-67 staining, according to Tuominen *et al*.^64^ Collagen quantity in images from Masson´s trichrome staining was determined by HSI-thresholding in NIS Elements AR 3.10 (Nikon Instruments), and the same program was also applied to quantify the extent of necrosis in H&E-stained slides, and for determining CD31^+^-microvessels, essentially as described in ref. 65. Images were collected in a random systematic pattern, due to lack of hot spots in many of our tumors.

In images representing FAP-1 and MPO stained sections, a semi-quantitative cell density scoring system was devised: **a)** FAP-1: **0** = (0–1 positive cells)/field; **1** = (2–10 positive cells)/field; **2** = (11–25 positive cells)/field; and **3** = (>25 positive cells)/field; **b)** MPO: **0** = (0–5)% positive staining/tumor area; **1** = (5–25)%; **2** = (26–50)%; **3** = (>50%). Scoring-system for intratumoral infiltration of Arg-1^+^ cells was (**0** = (0–5)% positive staining/tumor area; **1** = (5–25)%; **2** = (26–50)%; **3** = (>50%) and for iNOS (**0** = (0–1)%; **1** = (2–5)%; **2** = (6–15)%, **3** = (>16)), whereas peritumoral infiltration for the latter two markers was scored as: **0** = (0–5)%; **1** = (5–25)%; **2** = (26–50)%; **3** = (>50%). Results represent average from two researchers scoring independently. Discordance in results bigger than one were solved with a discussion and rescoring.

### CAF fate and CMFDA staining

To track implanted human CAFs in tumors, we identified CAFs in resected tumor tissues by two independent labelling methods. Firstly, CAFs in monolayers were fluorescence labelled, based on intracellular accumulation of cell tracker Green CMFDA (5-chloromethylfluorescein diacetate, Molecular Probes). To this end, CAFs were allowed to internalize CMFDA (5 μM) for 30 min, according to the manufacturer’s instruction, and 24 hours before implantation. CMFDA-labelled CAFs (1 × 10^6^), were co-implanted with A549 cells (1 × 10^6^) and injected (s.c.) into mice. Xenograft tumors established in animals were collected at one, two and four weeks after implantation. Excised tumors were fixed in 4% PFA (4 °C), paraffin-embedded and sectioned with a cryostat. Sections were directly examined for the presence of CMFDA-containing CAFs in a fluorescence microscope. Alternatively, tumor sections were stained with human-specific anti-PDGFRα antibody for detection of implanted human CAFs. For quantitative determinations, PDGFRα^+^-cells were counted in (2–3) random, non-overlapping areas of tumors. Semi-quantitative scorings were performed on PDGFRα-stained tumors according to the following indications: (0 = no staining; 1 = weak staining; 2 = moderate; 3 = abundant). Scores per mouse represent average value for all inspected areas of one tumor.

### Statistical analysis

One-way ANOVA followed by least significant difference (LSD) for *post hoc* analysis was used for multiple comparison when data were normally distributed. Kruskal-Wallis test followed by LSD *post hoc* analysis was used when data failed normality test. Normality of the data was investigated by visual inspection of QQ plots. All statistical testing was performed using SPSS (version 23). Significance was set at p < 0.05.

## Additional Information

**How to cite this article:** Grinde, M. T. *et al*. Ionizing radiation abrogates the pro-tumorigenic capacity of cancer-associated fibroblasts co-implanted in xenografts. *Sci. Rep.* **7**, 46714; doi: 10.1038/srep46714 (2017).

**Publisher's note:** Springer Nature remains neutral with regard to jurisdictional claims in published maps and institutional affiliations.

## Figures and Tables

**Table 1:** Overview of primary antibodies, their species reactivity, suppliers and product information, host species, and antibody dilution used during immunohistochemistry staining.

Marker	Antibody	Species reactivity	Supplier and catalogue no.	Host species	Antibody dilution
Proliferation	Anti-Ki67	Human	Roche 790-4286	Rabbit	Prediluted
Endothelial cells/angiogenesis	Anti-CD31	Mouse, human, pig	Abcam Ab28364	Rabbit	1:75
Neutrophiles granulocytes	Anti-MPO	Mouse, human, rat	Life Technologies PA5-16672	Rabbit	1:100
M1-macrophages	Anti-iNOS	Mouse and rat	Abcam Ab15323	Rabbit	1:100
M2-macrophages	Anti-Arginase1	Mouse, rat, human	Santa Cruz Biotechnology, Sc-20150	Rabbit	1:200
Host fibroblasts	Anti-Fap1	Mouse, rat and human	Abcam Ab53066	Rabbit	1:100
Human CAFs	Anti-PDGFRα	Human	Cell Signaling D13C6	Rabbit	1:50
